# News from Societies

**Published:** 1955-11

**Authors:** 


### *News from Societies*

### CORNWALL CLINICAL SOCIETY

(October 1954 to August 1955)

*President:* T. M. Banham Esq., M.D., D.L.O.
*Hon. Secretaries:* Dr. J. D. Hardy; Dr. T. S. Wilson.
*Hon. Treasurer:* Dr. W. L. Stewart.

The Society had a most successful year. There was a full programme of clinical meetings, there being one meeting every month held alternately at the Royal Cornwall Infirmary and the Camborne-Redruth Miners' and General Hospital.

The following papers were read and cases demonstrated:

Dr. C. T. Andrews: "Home Care in Diabetes".
Dr. E. R. Hargreaves: "Epidemiology of Diabetes in Cornwall".
Dr. C. D. R. Pengell: "Cirrhosis of the Liver Patent Ductus Arteriosus Case for Diagnosis".
Mr. T. E. Rutter: "Recent Concepts of Gall Bladder Disease".
Dr. B. A. Gwynne Jenkins: "Achalasia of the Cardia; Coarctation of the Aorta".
Dr. J. A. G. Hair: "Radiotherapy".
Dr. T. A. B. Mason: "Some X-rays of interest".
Dr. W. H. St. John Brooks: "Some of the Newer Drugs".
Dr. A. E. Tinkler: "V.D. in Cornwall".
Mr. S. R. Adlington: "Case of Regional Ileitis A. N. Other case".
Dr. J. D. Hardy: "A Case of Endocrine Disturbance".
Mr. P. N. Simons: "Shock in Obstetrics".

The April meeting was amalgamated with the Annual B.M.A. Clinical Meeting.

The July meeting was entirely clinical in character and 20 cases and other demonstrations were presented by the Consultant Staff of the Royal Cornwall Infirmary. This was a particularly good meeting.

The attendance at the meetings was distinctly better than of latter years, averaging 30 to 50. (Considering, however, that 250 individual notices are sent out, there is clearly room for further improvement.) The "officials" like to think the increased enthusiasm was due to the interesting material presented, although possibly the provision of light alcoholic refreshments may have had its influence!

The Society's dinner and dance in December, at the Hotel Bristol, Newquay, was the first event of its kind run by the Society. This "meeting" was very well attended and voted an unqualified success by everybody.

### TORQUAY AND DISTRICT MEDICAL SOCIETY

1954–55 Session

The Society's activities have continued on the same scale as the previous session. Six new members have been elected and the total membership is now 130.

Speakers for the ordinary meetings were as follows:

Dr. C. A. Boucher: " Accidents at Home".
Dr. Stephen Taylor: "The Content of General Practice".
Dr. H. L. Marriott: "Recent Medical Advances of Practical Importance".
Dr. F. S. W. Brimblecombe: "Failure to Thrive in Infancy".
Sir George MacRobert: "Tropical Medicine in England in the Air Age".
Sir Clement Price-Thomas: "Cancer of the Lung".
Professor Ian Aird: "The Siamese Twin Operation".

The average attendance at seven ordinary meetings, including guests, was 45 (36 last year).

A clinical discussion opened by the President on tuberculosis and its management in this part of the country was attended by 26 members. The combined clinical meeting with the South-Western Branch of the B.M.A. was held as usual in January, but owing to the severe weather only 20 members attended.

A joint clinical discussion on Oral Sepsis was attended by 15 members of the Dental Society and 14 members of the Medical Society. The discussion was opened by excellent addresses by two members of each society.

The annual dinner and dance after the opening meeting was held at the Grand Hotel; 115 members and guests were present.

The 1954 summer meeting was held at St. Loyes College Exeter. Seventeen members were present and a most interesting address was followed by a tour of the rehabilitation departments.

The 1955 summer meeting was held before the annual general meeting—it being thought that an earlier date might attract more members. It took place at Exminster Hospital where an address on certain forms of therapy in mental disease was followed by a tour of the departments and gardens. Nineteen members were present.

### BRISTOL MEDICO-CHIRURGICAL SOCIETY

*President:* Dr. Percy Phillips.

*Hon. Secretary:* Dr. B. E. McConnell.

The full programme has not yet been arranged but the clinical meeting will be at Frenchay Hospital on 9th November, and Sir Lionel Whitby will address the Society on **"Anaemia of Pregnancy"**, on 8th February, 1956.

### B.M.A. (Bristol Division)

*Chairman:* Dr. L. G. Robertson.

*Hon. Secretaries:* Dr. A. S. Anderson and Dr. Sarah Walker.

1955

Saturday, 1st October, dep. 4.45 p.m.: Visit to Wildfowl Trust, Slimbridge, Gloucestershire (Coach party).

Wednesday, 19th October, 8.30 p.m.: Professor J. M. Webster, M.A., B.SC., M.B., CH.B., M.D., F.R.C.S., Director of the West Midland Forensic Science Laboratory (Pathologist to the Home Office), **"Forensic Science".** (B.M.A. Popular Lecture: Members may invite a friend.) Main Physics Lecture Theatre, Royal Fort, University of Bristol.

1956

Wednesday, 18th January, 8.30 p.m.: Miss E. Ralph, Archivist to City of Bristol, **"Old Bristol".** (Members may invite a friend.) Main Physics Lecture Theatre, Royal Fort.

*Wednesday, 16th May, 8.30 p.m.: Annual General Meeting. Small Physics Lecture Theatre, Royal Fort.

*Summer: Social event—for members and friends. To be arranged.

Wednesday, 20th June, 8.30 p.m.: Clinical meeting. Bristol Royal Infirmary.

*Friday, 22nd June, 4.0 p.m.: B.M.A. Reception for newly-qualified doctors (Bristol University)—Garden Hall, Royal Fort, Bristol.

* Provisional dates.

### WEST OF ENGLAND CHILD HEALTH GROUP

*Chairman:* Dr. Greta Hartley.

*Hon. Secretary:* Dr. R. C. Wofinden.

Friday, 23rd September, 8.30 p.m.: Dr. Beryl Corner, F.R.C.P., Consultant Paediatrician, **"Paediatrics in the U.S.A.".** Small Physics Lecture Theatre, Royal Fort.

Friday, 14th October, 8.15 p.m.: Members of the staff of the Children's Hospital, Clinical meeting. Out-Patient Dept., Children's Hospital.

Thursday, 10th November, 5.30 p.m.: R. E. Horton, M.B.E., F.R.C.S., Department of Surgery, University of Bristol, **"The present status and future of intracardiac surgery".** Small Physics Lecture Theatre, Royal Fort.

December (date to be announced later), 8.30 p.m.: W. Hobson, B.SC., M.D., D.P.H., Professor of Social and Industrial Medicine, University of Sheffield, **"Congenital Defects".** Small Physics Lecture Theatre, Royal Fort.

Thursday, 12th January, 8.30 p.m. Mrs. Janet Bodman, William Budd Health Centre; Mrs. Veronica Hope, Child Guidance Clinic; Miss Irene Westmacott, Children's Hospital; **"Psychiatric Social Work in the field of Child Health".** Out-Patient Dept., Children's Hospital.

Wednesday, 29th February, 5.30 p.m.: Dr. I. R. Gordon, Senior M.O., M. and C.W. Services, County of Somerset, **"Rural Problems in Child Health".** Out-Patient Dept., Children's Hospital.

Thursday, 29th March, 8.30 p.m.: H. Paul, M.D., B.CH., D.P.H., Medical Officer of Health, County Borough of Smethwick. Subject to be announced later. Small Physics Lecture Theatre, Royal Fort.

Friday, 27th April, 8.30 p.m.: Dr. W. Lumsden Walker, Medical Superintendent, Hortham Hospital; Dr. H. Temple Phillips, Chief Assistant M.O., Health Department, **"Some Problems in Mental Deficiency".** Small Physics Lecture Theatre, Royal Fort.

May, Friday afternoon (date to be announced later): Field visit to Avonmouth Docks. (Limited numbers.)

### BRISTOL SOUTH MEDICAL GROUP

*Hon. Secretary:* Dr. D. M. Cameron.

The Group meets on the first Sunday of the month from October to May in the Board Room of the Bristol General Hospital at 8.0 p.m. for discussions on clinical and medico-political matters.

### BRISTOL NORTH-WEST GROUP

*Hon. Secretary:* Dr. Maxwell.

This Group has been formed by the amalgamation of the Westbury and Gloucester Road Groups.

### BATH CLINICAL SOCIETY

*President:* Dr. H. Gibson.
*Hon. Secretary:* Dr. D. W. Pugh.

At the October meeting there will be a combined discussion on **"Brucellosis"**, of which disease there has been an outbreak in Wiltshire. At the meeting in November clinical cases will be shown.

### B.M.A. GLOUCESTERSHIRE BRANCH

*President:* Dr. H. S. K. Lowry.
*Hon. Secretaries:* Dr. E. W. Hyde, Dr. R. H. Ellis.
October: Presidential address by Dr. H. S. K. Lowry.
November: Clinical meeting at Cheltenham General Hospital.
December: B.M.A. lecture by Dr. E. A. Bennett on Medical Hypnotism.
January: Meeting at the New Health Centre in Cheltenham with a discussion on its working.
February: Paper by Dr. G. R. Fearnley: **"The Management of the Rheumatoid Case"**.
March: to be arranged.
April: Clinical meeting at Gloucestershire Royal Infirmary.
May: Annual general meeting and a Paper on a Dermatological Subject by Dr. R. E. Bowers.

### THE DEVON AND EXETER MEDICO-CHIRURGICAL SOCIETY

*President:* Dr. Ronald S. Coldrey.
*Hon. Secretary:* Dr. Charles Seward.

1955

20th October, at 8.15 p.m.: Presidential address by Dr. Ronald Coldrey, **"The Changing Pattern"**.

10th November, 4.15 p.m.: A combined meeting with B.M.A. Commencing with tea at 3.45 p.m. Dr. J. H. Sheldon, F.R.C.P., President of International Assn. of Gerontology, **"Some Problems of Old Age"**. (South-Western Dinner.)

1st December, 8.15 p.m.: Clinico-pathological demonstration.

29th December, 8.15 p.m.: H. Dendy Moore, Esq., F.R.C.S., **"The use of the radio-opaque medium Diodone in Surgical Diagnosis and Treatment"**.

1956

19th January, 3.30 p.m.; Clinical Meeting. Commencing with Tea.

16th February, 8.15 p.m.: Dr. Edward Cullinan, F.R.C.P., **"The Diagnosis of Jaundice"**.

8th March, 8.15 p.m.: Clinico-pathological demonstration.

22nd March, 8.15 p.m.: Symposium—**"The Church and Medicine"**. Principal Speakers—The Bishop of Crediton and Dr. L. N. Jackson, M.C.

Saturday, 21st April, 2.30 p.m.: Clinical meeting at the Princess Elizabeth Orthopaedic Hospital, concluding with tea.

### TORQUAY AND DISTRICT MEDICAL SOCIETY

1955

27th October: Opening meeting, Dinner and dance. Guest speaker: Sir John Charles.
10th November: Dr. J. E. Cates, **"Advances in Theraputics"**.
*24th November: Combined Clinical Meeting with B.M.A.
8th December: Prof. S. B. Bedson, **"Viruses, and some Diseases they cause in man"**.

1956

26th January: Mr. D. C. Bodenham, **"Some Problems of Burn Injuries"**.
9th February: Dr. R. Sessions Hodges, **"Electro-encephalography"**.
*8th March: Clinical Discussion. Opened by Mr. J. MacPherson.
29th March: Dr. C. A. H. Watts, **"A Study of Depressive States in General Practice"**.
26th April: Prof. J. M. McMichael, **"Some After-thoughts on Cardiac Surgery"**.
*24th May: Annual general meeting.

All ordinary Meetings are held at the Torbay Hospital at 8.30 p.m. Opening meeting at 4.0 p.m.

* Time and Place to be settled later.

### WEST PENWITH MEDICAL SOCIETY

*President:* Dr. L. P. Lockhart.
*Hon. Secretary:* Dr. D. E. Truscott.

Meetings are held on the third Thursday in each month and are devoted alternately to clinical cases and the reading of papers.

The annual dinner of the Society will be held at the Porthminster Hotel, St. Ives, on 28th October, 1955.

### SOUTH SOMERSET MEDICAL CLUB

*Hon. Secretary:* Dr. P. P. Fox, Public Health Dept., Preston Road, Yeovil.

1955

September: Talk by Prof. Neale on **"Paediatricians' Problems Met in General Practice"**.

November: Annual general meeting and a discussion on Immunization.

1956

January: Dinner and dance.

March and July Meetings: details have not yet been settled.

### PLYMOUTH MEDICAL SOCIETY

*President:* Dr. L. O. Lindsay.

*Hon. Secretary:* Dr. J. W. M. Owen.

1955

Friday, 14th October, 8.30 p.m.: Dr. Michael Ward, **"Ascent of Everest"**.

Wednesday, 19th October: Annual dinner and dance.

Friday, 28th October: Clinical evening.

Friday, 4th November: Film, **"A Two-Year-Old goes to Hospital"**. Discussion, introduced by Dr. Jolly.

Friday, 18th November: Prof. J. M. Webster, C.B.E., M.D., F.R.C.S. (ED.), **"A General Review of the History of Forensic Science"**.

Friday, 2nd December: Prof. D. W. Smithers, M.D., M.R.C.P., **"Clinical Cancer Research"**.

Friday, 16th December: Clinical evening.

1956

Friday, 20th January: Dilwyn M. G. Thomas, Esq., F.R.C.S., **"Surgery of Primary Intra-thoracic Tuberculosis"**.

Friday, 3rd February: Clinical evening at the Royal Naval Hospital by courtesy of Surgeon Rear-Admiral A. A. Pomfret, O.B.E., Q.H.S.

Friday, 17th February: Prof. R. A. McCance, F.R.S., **"Food for Adventure"**.

Friday, 2nd March: **"Any Questions"**, a medical programme.

Wednesday, 14th March, 2.30 p.m.: Visit to Mount Gold Orthopaedic Hospital.

### UNIVERSITY OF BRISTOL

1955

16th October, 10.30 a.m.: Mr. W. G. MacGregor, Professor G. Gordon Lennon, Mr. Ashton Miller, Dr. R. J. Irving-Bell, Dr. F. N. Boxall; Bristol Royal Infirmary: Dept. of Medicine. **"Infertility"**.

23rd October, 10.30 a.m.: Mr. A. L. Eyre-Brook, **"Minor Ailments of the Foot"**. Bristol Royal Infirmary: Dept. of Surgery.

30th October, 10.30 a.m.: Professor A. V. Neale, Clinical Round. Children's Hospital.

6th November, 10.30 a.m.: Mr. J. Angell James, **"Catarrh"**. Bristol Royal Infirmary: Dept. of Surgery.

13th November, 10.30 a.m.: Mr. D. C. Bodenham, **"Prevention and Treatment of Burns in the Home"**. Bristol Royal Infirmary: Dept. of Surgery.

20th November, 10.30 a.m.: Dr. T. G. Faulkner Hudson, **"Some New Hazards at Work"**. Bristol Royal Infirmary: Dept. of Medicine.

27th November, 10.30 a.m.: Dr. Beryl Corner, Clinical Round. Children's Hospital.

